# Synthesis and Cytotoxic Evaluation of Some Novel SulfonamideDerivativesAgainst a Few Human Cancer Cells

**Published:** 2011

**Authors:** Mina Mirian, Afshin Zarghi, Sedighe Sadeghi, Parisa Tabaraki, Mojdeh Tavallaee, Orkideh Dadrass, Hojjat Sadeghi-aliabadi

**Affiliations:** a *Isfahan Pharmaceutical Research Center, School of Pharmacy, Isfahan University of Medical Sciences, Isfahan, Iran.*; b *School of Pharmacy, ShahidBeheshti University of Medical Sciences (M. C), Tehran, Iran.*; c *School of Pharmacy, Azad University, Tehran, Iran.*

**Keywords:** Sulfonamides, MTT assay, MDA-MB-468, HeLa, MCF-7

## Abstract

Sulfonamides are the first effective chemotherapeutic agents used for several years to cure or prevent systemic bacterial infections. In addition, this agents showed anti-carbonic anhydrase and cause cell cycle perturbation in the G_1_ phase, disruption of microtubule assembly, suppression of the transcription activator Nf-Y, angiogenesis and matrix metalloproteinase (MMP). In recent years, novel synthesized sulfonamides have been introduced as antitumor, antiviral and anti-inflammatory agents.

In this paper, the cytotoxic effects of 8 synthesized sulfonamides were investigated by MTT assay on HeLa, MDA-MD-468 and MCF-7 cancer cell lines. Human cancer cells were cultured and passaged in RPMI-1640 medium. Cells incubated in 96-well plates in a concentration of 1 × 10^5^ cells/mL for 24 h, and then logarithmic concentrations (0.1 μm, 1 μm, 10 μm, 100 μm, 1mM) of each drug were prepared, added to the plates and incubated for 72 h. Cell survival was then determined using ELISA plate reader in 540 nm applying MTT assay.

All tested sulfonamides showed cytotoxic effect on HeLa and MCF-7 cells in the concentration range of 100-1000 μm. These sulfonamides were cytotoxic against MDA-MB-468 cell line at a concentration of 10-100 μm and reduced the cell survival less than 50%.

According to the results calculated IC_50_’s were as following: MDA-MB-468 < 30 μm; MCF-7 < 128 μm and HeLa< 360 μm. In conclusion, some tested sulfonamides had good cytotoxic effects against breast cancer cells, MDA-MB-468 and further investigations are needed to confirm their effects against other cells.

## Introduction

Sulfonamide was the first antimicrobial drug that its chemical moiety is also present in other medications which are not antimicrobials, including thiazide diuretics (including hydrochlorothiazide), loop diuretics (including furosemide), some COX-2 inhibitors (*e.g. *celecoxib) and also utilized in the treatment of inflammatory bowel disease (*e.g. *Sulfasalazine). Recently, sulfa drugs were introduced as protease inhibitors; therefore, they can be used as anticancer, anti-inflammatory and antiviral agents (1). Some of sulfonamide derivatives with photodynamic activities used against nasopharyngeal carcinoma cells and their anti-tumor and anti-angiogenesis activities were shown in a dose dependent manner (2). In a study to find new anti-tumor agents, a series of sulfonamide hydroxamic acids and anilides have been synthesized and evaluated as histone deacetylase (HDAC) inhibitors which can induce hyper-acetylation of histones in human cancer cells. Bouchain*et al. *showed that synthesized sulfonamides selectively inhibit proliferation, blocks the cell cycle and induce apoptosis in human cancer cells but not in normal cells (3, 4). E7070, [N-(3-Chloro-7-indolyl)-1,4-benzenedisulfonamide] is a novel sulfonamide anticancer agent currently in phase II clinical development for the treatment of solid tumors (5). This compound, indisulam (6), strongly inhibits carbonic anhydrase, a critical enzyme involved in many physiological processes and whose association with cancer became obvious in the last period, is a cell-cycle inhibitor that arrests the cell cycle at the G1/S transition (7, 8). In an attempt to find new antibacterial and anti-inflammatory compounds, a series of sulfonamides have been synthesized and their biological effects including antibacterial, anti-inflammatory and also cytotoxic properties were evaluated (9).

## Experimental

*General*

All chemicals and solvents used in this study were purchased from Merck AG and Aldrich except for doxorubicin which obtained from (David Bull Labs, UK). Melting points were determined with a Thomas–Hoover capillary apparatus. Infrared spectra were acquired using a Perkin Elmer Model 1420 spectrometer. A Bruker FT-500 MHz instrument (Bruker Biosciences, USA) was used to acquire ^1^HNMR spectra with TMS as internal standard.

Chloroform-D and DMSO-D_6_ were used as solvents. Coupling constant (*J*) values are estimated in hertz (Hz) and spin multiples are given as s (singlet), d (double), t (triplet), q (quartet), m (multiplet), and br (broad). Low-resolution mass spectra were acquired with a MAT CH5/DF (Finnigan) mass spectrometer that was coupled online to a Data General DS 50 data system. The mass spectral measurements were performed on a 6410 Agilent LCMS triple quadruple mass spectrometer (LCMS) with an electrospray ionization (ESI) interface. Microanalyses, determined for C and H, were within ± 0.4% of theoretical values.

*Chemistry*

**Figure 1:**
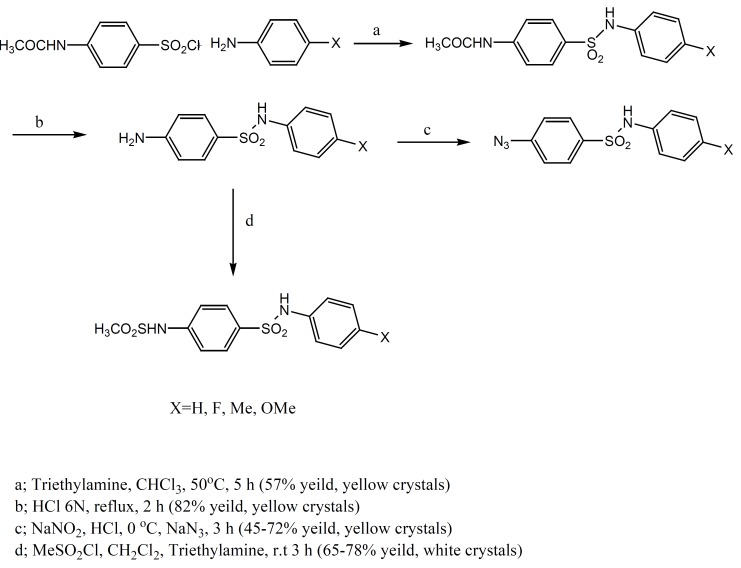
Schematic representation of synthesis of sulfonamide derivatives

Sulfonamides used in this work were synthesized according to Figure 1.

*General procedure for preparation of 4- acetamido -4- substituted- phenyl- benzenesulfonamide 1*

15 mmol of 4-acetamidobenzene sulfonyl chloride was added to a solution of 4-substituted-aniline (15 mmol) and triethyl amine (5 mL) in chloroform (25 mL) and stirred at 25°C for 15 min. Then the mixture was heated gently up to 55-60°C. After 5 h, the organic solvent was evaporated and 100 mL cold water was added. The produced precipitate was filtered and washed with water and recrystallized in ethanol (yields: 65-87%).

*4-Acetamido-4-phenyl-benzenesulfonamide 1a*

Yield: 87%; white crystalline powder; mp = 203-204°C; IR (KBr): ν (cm^-1^) 3310, 3200 (NH), 1670 (C=O), 1320, 1150 (SO_2_); LC-MS (ESI +) m/z: 291.1 (M + 1) (100).

*4-Acetamido -4-fluorophenyl-benzenesulfonamide 1b*

Yield: 67%; white crystalline powder; mp = 188-190°C; IR (KBr): ν (cm^-1^) 3320, 3200 (NH), 1680 (C=O), 1320, 1140 (SO_2_); LC-MS (ESI+) m/z: 309.1 (M + 1) (100).

*4-Acetamido-4-methoxyphenyl benzenesulfonamide 1c*

Yield: 65%; cream crystalline powder; mp = 200-201°C; IR (KBr): ν (cm^-1^) 3300, 3200 (NH), 1650 (C=O), 1300, 1120 (SO_2_); LC-MS (ESI+) m/z: 321.1 (M + 1) (100).

*4-Acetamido-4-methylphenyl-benzenesulfonamide 1d*

Yield: 65%; cream crystalline powder; mp = 200-201°C; IR (KBr): ν (cm^-1^) 3300, 3200 (NH), 1660 (C=O), 1300, 1100 (SO_2_); LC-MS (ESI+) m/z: 305.1 (M + 1) (100).

*General procedure for preparation of 4-amino-4-substituted-phenyl-benzenesulfonamide 2*

To 12 mmol of 1, 10 mL 6N HCl was added and refluxed for 6 h. after this time, the solution was neutralized by 25% sodium hydroxide; the obtained product was filtered, washed and crystallized by ethanol (yields: 79-90%).

*4-Amino-4-phenyl-benzenesulfonamide 2a*

Yield: 82%; yellow crystalline powder; mp = 195-196°C; IR (KBr): ν (cm^-1^) 3380, 3320 (NH_2_), 3200(NH), 1310, 1150 (SO_2_); LC-MS (ESI+) m/z: 249.1 (M + 1) (100)

*4-Amino-4-fluorophenyl-benzenesulfonamide 2b*

Yield: 81%; white crystalline powder; mp = 165-167°C; IR (KBr): ν (cm^-1^) 3400, 3320 (NH_2_), 3200 (NH), 1300, 1140 (SO_2_); LC-MS (ESI+) m/z: 267.1 (M + 1) (100).

*4-Amino-4-methoxyphenyl-benzenesulfonamide 2c*

Yield: 90%; cream crystalline powder; mp = 195-196°C; IR (KBr): ν (cm^-1^) 3400, 3300, (NH_2_), 3200 (NH), 1340, 1100 (SO_2_); LC-MS (ESI+) m/z: 279.1 (M + 1) (100).

*4-Amino-4-methylphenyl-benzenesulfonamide 2d*

Yield: 79%; yellow crystalline powder; mp = 190-191°C; IR (KBr): ν (cm^-1^) 3350, 3250, (NH_2_), 3200 (NH), 1310, 1100 (SO_2_); LC-MS (ESI+) m/z: 263.1 (M + 1) (100).

*General procedure for preparation of 4-azido-4-substituted-phenyl-benzenesulfonamide 3*

To 12 mmol of 2, 6 mL 10N HCl and 10 mL water were added and the mixture stirred for 1 h. Then a solution of 13 mmol sodium nitrite in 5 mL water was added drop wise. After 15 min, a solution of sodium azide (13 mmol) in water (5 mL) was added and the mixture was stirred for 1 h. The precipitated product was filtered, washed and crystallized by ethanol (yields: 83-96%).

*4-Azido-4-phenyl-benzenesulfonamide 3a*

Yield: 90%; white crystalline powder; mp = 95°C; IR (KBr): ν (cm^-1^) 3200(NH), 2100 (N_3_) 1300, 1150 (SO2); ^1^HNMR (CDCl_3_): δ ppm 6.46 (s, 1H, NH), 7.07-7.29 (m, 7H, phenyl and 4-azidophenyl H_3_ and H_5_), 7.77 (d, 2H, 4-azidophenyl H_2_ and H_6_ , *J *= 8.7 Hz) ;LC-MS(ESI+) m/z : 275.1 (M + 1) (100).

*4-Azido-4-fluorophenyl-benzenesulfonamide 3b*

Yield: 96%; white crystalline powder; mp = 97°C; IR (KBr): ν (cm^-1^) 3200(NH), 2050 (N_3_) 1300, 1120 (SO_2_); ^1^HNMR (CDCl_3_): δ ppm 6.64 (s, 1H, NH), 7.00 (t, 2H, 4-fluorophenyl H_3_ and H_5_), 7.06-7.10 (m, 4H, 4-azidophenyl H_3_ and H_5_ and 4-fluorophenyl H_2_ and H_6_), 7.73 (d, 2H, 4-azidophenyl H_2_ and H_6 _, *J *= 8.6 Hz) ; LC-MS(ESI+) m/z: 293.1 (M + 1) (100).

*4-Azido-4-methoxyphenyl-benzenesulfonamide 3c*

Yield: 83%; cream crystalline powder; mp = 74°C; IR (KBr): ν (cm^-1^) 3200(NH), 2030 (N_3_) 1300, 1120 (SO_2_); ^1^HNMR (CDCl_3_): δ ppm 3.81 (s, 3H, OCH_3_),6.23 (s, 1H, NH), 6.82 (d, 2H, 4-methoxyphenyl H_2_ and H_6_, *J *= 8.8 Hz), 7.01 (d, 2H, 4-methoxyphenyl H_3_ and H_5_, *J *= 8.8 Hz), 7.08 (d, 4H, 4-azidophenyl H_3_ and H_5_ , *J *= 8.7 Hz), 7.71 (d, 2H, 4-azidophenyl H_2 _and H_6_ , *J *= 8.7 Hz) ;LC-MS (ESI+) m/z: 305.1 (M + 1) (100).

*4-Azido-4-methylphenyl-benzenesulfonamide 3d*

Yield: 94%; white crystalline powder; mp = 79-80°C; IR (KBr): ν (cm^-1^) 3180(NH), 2040 (N_3_) 1300, 1120 (SO_2_); ^1^HNMR (CDCl_3_): δ ppm 2.33 (s, 3H, CH_3_),6.43 (s, 1H, NH), 6.98 (d, 2H, 4-methylphenyl H_2_ and H_6_, *J *= 8.2 Hz), 7.07 (d, 2H, 4-methylphenyl H_3_ and H_5_, *J *= 8.2 Hz), 7.11 (d, 4H, 4-azidophenyl H_3_ and H_5_ , *J *= 8.6 Hz), 7.75 (d, 2H, 4-azidophenyl H_2 _and H_6_ , *J *= 8.6 Hz) ; LC-MS(ESI+) m/z: 289.1 (M + 1) (100).

*General procedure for preparation of 4-methyl sulfamido-4-substituted-phenyl-benzenesulfonamide 4*

To 10 mmol of 2, 100 mL dichloromethane and 6 mL triethyl amine were added. Then, 15 mmolmethanesulfonylchloride was added drop wisely during 30 min and the mixture stirred for 3 h at room temperature. After the completion of reaction, the organic solvent was evaporated and 100 mL cold water was added and stirred vigorously. The produced precipitate was filtered and washed with water and recrystallized in ethanol (yields: 70-83%).

*4-Methyl sulfamido-4-phenyl-benzenesulfonamide 4a*

Yield: 72%; white crystalline powder; mp = 160-161°C; IR (KBr): ν (cm^-1^) 3200(NH), 1340, 1150 (SO_2_); ^1^HNMR (CDCl_3_): δ ppm 2.84 (s, 3H, SO_2_Me), 6.89-7.06 (m, 5H, phenyl) 7.15 (4-methylsulfamidophenyl H_3_ and H_5_, *J *= 8.7 Hz), 7.58 (d, 2H, 4- methylsulfamidophenyl H_2_ and H_6_ , *J *= 8.7 Hz), 9.43 (s, 1H, NH), 9.89 (s, 1H, NH) ; LC-MS (ESI+) m/z: 327.2 (M + 1) (100).

*4-Methyl sulfamido-4-fluorophenyl-benzenesulfonamide 4b*

Yield: 70%; white crystalline powder; mp = 175-176°C; IR (KBr): ν (cm^-1^) 3200(NH), 1340, 1100 (SO_2_); ^1^HNMR (CDCl_3_): δ ppm 2.83 (s, 3H, SO2Me), 6.72 (t, 2H, 4-fluoro phenyl H3 and H5), 6.94 (d, 2H,4-fluorophenyl H_2_ and H_6_, *J *= 8.7 Hz)),7.14 (4-methyl sulfamidophenyl H_3_ and H_5_, *J *= 8.8 Hz), 7.51 (d, 2H, 4- methyl sulfamidophenyl H_2_ and H_6 _, *J *= 8.8 Hz), 9.43 (s, 1H, NH), 9.91 (s, 1H, NH) ; LC-MS (ESI+) m/z: 345.2 (M + 1) (100).

*4-Methyl sulfamido-4-methoxyphenyl-benzenesulfonamide 4c*

Yield: 83%; cream crystalline powder; mp = 181-182°C; IR (KBr): ν (cm^-1^) 3200(NH),1300, 1120 (SO_2_); ^1^HNMR (CDCl_3_): δ ppm 2.10 (s, 3H, CH_3_),2.82 (s, 3H, SO_2_Me), 6.81 (d, 2H, 4-methylphenyl H_2_ and H_6_, *J *= 8.5 Hz), 6.85 (d, 2H, 4-methyl phenyl H_3_ and H_5_, *J *= 8.5 Hz),7.12 (4-methyl sulfamidophenyl H_3_ and H_5_, *J *= 8.8 Hz), 7.52 (d, 2H, 4- methyl sulfamidophenyl H_2_ and H_6_ , *J *= 8.8 Hz), 9.22 (s, 1H, NH), 9.87 (s, 1H, NH) ; LC-MS (ESI+) m/z: 341.2 (M + 1) (100).

*4-Methyl sulfamido-4-methylphenyl-benzenesulfonamide 4d*

Yield: 70%; cream crystalline powder; mp = 193-194°C; IR (KBr): ν (cm^-1^) 3200(NH), 1340, 1100 (SO_2_); ^1^HNMR (CDCl_3_): δ ppm 2.78 (s, 3H, SO_2_Me), 3.63 (s, 3H, OCH_3_), 6.60 (d, 2H, 4-methoxy phenyl H_2 _and H_6_, *J *= 8.9 Hz), 6.91 (d, 2H, 4-methoxy phenyl H_3_ and H_5_, *J *= 8.9 Hz),7.17 (4-methyl sulfamidophenyl H_3_ and H_5_, *J *= 8.8 Hz), 7.54 (d, 2H, 4- methyl sulfamidophenyl H_2_ and H_6_ , *J *= 8.8 Hz), 8.98 (s, 1H, NH), 9.88 (s, 1H, NH) ; LC-MS (ESI+) m/z: 357.2 (M + 1) (100).

*General procedure of compounds preparation for biological studies*

To prepare 10 mM stock solution of each compounds, the appropriate amounts were dissolved in 1 mL of Tween 80 (20%) and other serial dilution were prepared from this stock solution, using PBS.

*Biological studies*

*Cell lines *HeLa (Human cervix carcinoma), MCF-7 (Human Caucasian/ breast cancer cells), MDA-MB-468 (Human breast adenocarcinoma) cell lines were purchased from Pasture Institute of Iran in Tehran. They were grown in RPMI-1640 [each 500 mL of RPMI-1640 was supplemented with 10% of fetal calf serum, 5 mL of penicillin/streptomycin (50 IU mL^-1^ and 500 µ g mL^-1^ respectively), NaHCO_3_ (1 g) and 5 mL of L-glutamine (2 mM)]. Completed media was sterilized through 0.22µ m microbiological filters after preparation and kept at 4°C before using. 

*MTT-based cytotoxicity assay*

The cytotoxic effects of synthesized compounds against previously mentioned human tumor cell lines were determined by a rapid colorimetric assay, using 3-(4, 5-dimethylthiazol-2-yl)-2, 5-diphenyl tetrazolium bromide (MTT) and compared with untreated controls (8). This assay is based on the metabolic reduction of soluble MTT by mitochondrial enzyme activity of viable tumor cells, into an insoluble colored formazan product, which can be measured spectrophotometrically after being dissolute in DMSO. Briefly, 200 µL of cells (1 × 10^5^ cells/mL) were seeded in 96-well microplates and incubated for 24 h (37°C, 5% CO_2_ air humidified). Then, 20 µl of final concentration of each compound was added and incubated for another 72 h in the same condition. Doxorubicin was used as a positive control. Cell survival was determined as already mentioned (11).

*Statistical analysis*

All results are expressed as mean ± SD of at least three experiments. P*-*values for significance were determined using the two-tailed Student›s t-test. Significance was assumed at 5% level.

## Results and Discussion

A good relationship between the absorbance and the number of cells was observed for HeLa, MDA-MB-468 and MCF-7 cell lines, (r^2^ = 0.9407, 0.8695 and 0.9907, respectively). Intraday and interday variations for all standard curves were acceptable (%CV<15). Taxol (2 mg/mL), a known cytotoxic drug, significantly inhibited the proliferation of all tested cell lines as a positive control to less than 20%. Drugs were considered cytotoxic where cell viability decreased to less than 50%.

*Cytotoxic effect of sulfonamide derivatives against HeLa cell*

**Figure 2:**
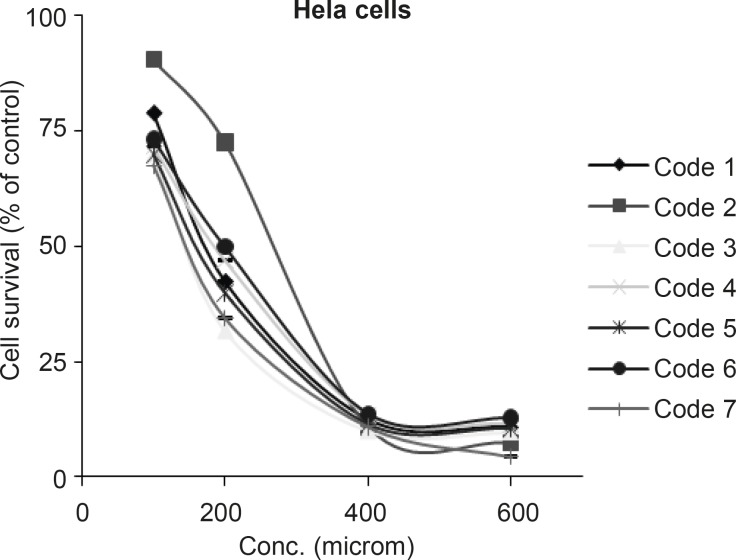
Dose response curve for HeLa cells following 72 h continuous exposure to 7 different compounds (n = 3), used for IC50 determination. Compound codes are mentioned in Table 1

All tested sulfonamides used in this research in the concentration range of 100-1000 µM on HeLa cell line showed cytotoxic effect and reduced the cell survival to < 50%. To determine the exact IC_50’s_, HeLa cells were exposed to different concentrations (100-600 μM) of compounds. Results are shown in Figure 2.

*Cytotoxic effect of sulfonamide derivatives against MDA-MB-468 cell*

**Figure 3:**
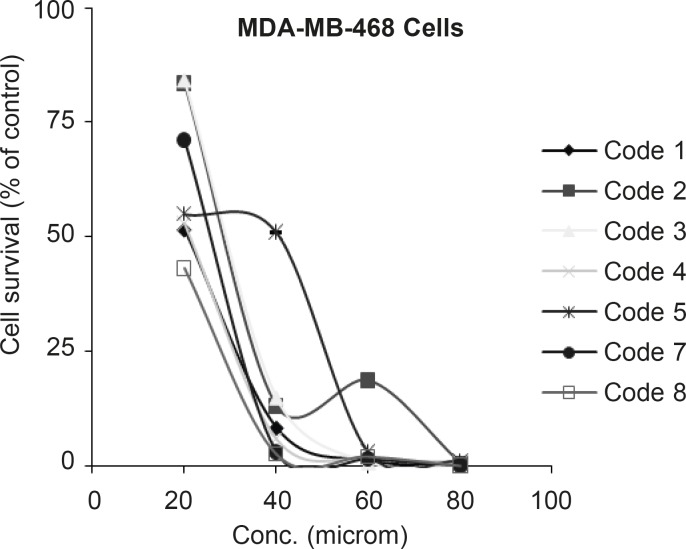
Dose response curve for MDA-MB-468 cells following 72 h continuous exposure to 7 different compounds (n = 3), used for IC50 determination. Compound codes are mentioned in Table 1

In the case of MDA-MB-468 cells, all tested compounds in the concentration range of 10-100 µM were cytotoxic and IC_50’s_ were determined as illustrated in Figure 3.

*Cytotoxic effect of sulfonamide derivatives against MCF-7 cell*

**Figure 4:**
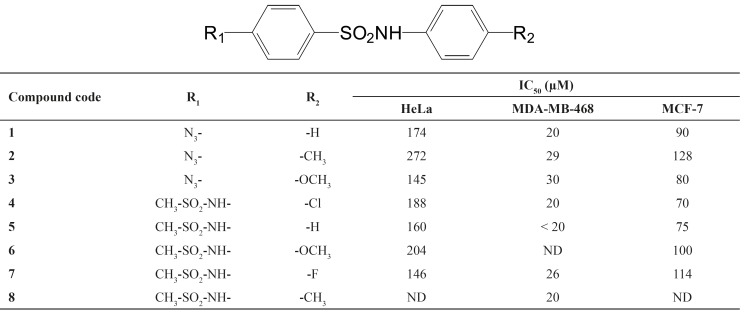
Dose response curve for MCF-7 cells following 72 h continuous exposure to 7 different compounds (n = 3), used for IC50 determination. Compound codes are mentioned in Table 1.

Tested sulfonamides in the concentration rang of 100-1000 µM on MCF-7 cell line showed cytotoxic effect and IC_50’s_ were determined as seen in Figure 4.

All calculated IC_50’s_ of synthetic compounds against tested cell lines are shown in Table 1 (p < 0.001).

**Table 1:** Synthesized compounds and their IC_50_’s against tested cell lines (n = 3).

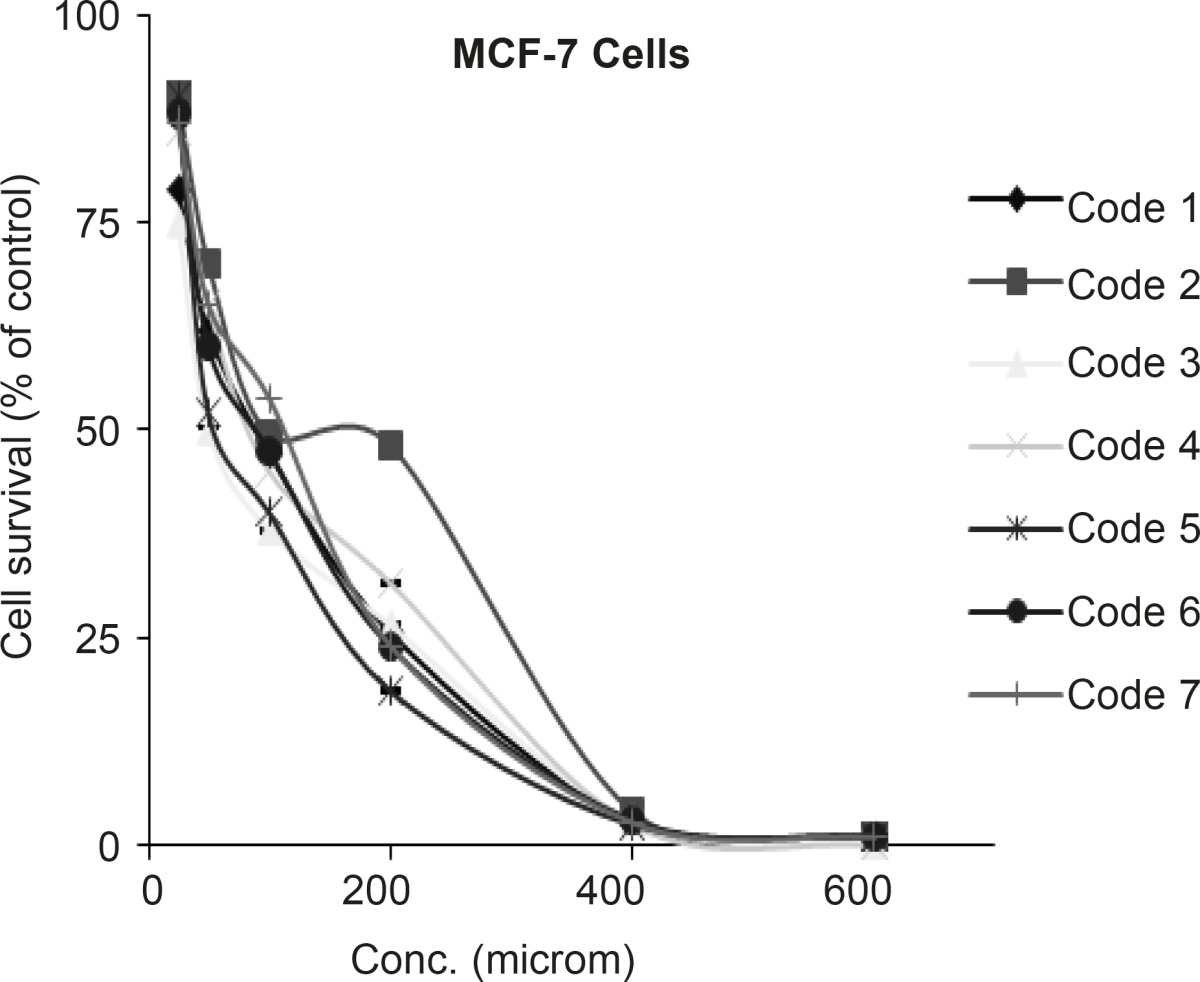

Sulfonamides are usually used in prevention and treatment of bacterial infections in human, but their importance in the field of cancer treatment is considered in recent years. Researchers show that these compounds have multi-effects, in addition to their anti-microbial properties (12).

New sulfonamides and their related derivatives have shown remarkable anti-tumor activities *in-vivo *and *in-vitro *that introduces them as a suitable candidate for preventing proliferation treatment of tumor cells in different kind of cancers (12).

Sulfonamides, through inhibiting protease activity, are introduced as anti-cancer, anti-virus and anti-inflammation agents. These compounds also prevent the activity of matrix metalloproteases (MMP) and transformed enzyme TNF-*α *(TACE); therefore, they are effective in pathophysiology of most of diseases such as arthritis, metastatic tumor and bacterial meningitis, simultaneously (13).

**Figure 5:**
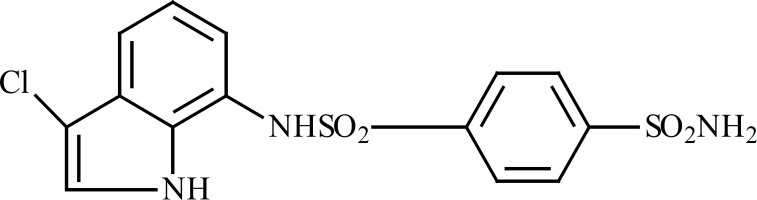
Chemical structure of E7070; a novel synthesized sulfonamide with anticancer effects

E7070 (Figure 5) is one of the sulfonamides that its cytotoxic effect has been evaluated (12). The chemical structure of E7070 is similar to our synthesized compounds; particularly (phenyl-NH-SO2-phenyl) moiety in E7070 has been repeated in our compounds.

Previous studies have been revealed that E7070 had an acceptable anti-tumor effect in cell lines including LX-1 of lung cancer, HCT-116 from colorectal (14) and A549 from lung cancer (13). This compound exerts its effect by interaction with cell cycle especially in S phase (15, 16) or by inhibiting carbonic anhydrase enzyme (17). The structural similarity of tested compound with E7070 may be responsible for their *in-vitro *cytotoxic effects.

According to the obtained data, (Table 1) these compounds showed most cytotoxic effects against breast cancer cell lines (MDA-MB-468 and MCF-7), with IC_50_ ≤ 30 μm and ≤ 100 μm, respectively. On the other hand, these compounds did not show significant cytotoxic effects against HeLa cells, from cervix cancer (145 μm ≤ IC_50 _≤ 360 μm). In agreement with these results, other researchers have shown that sulfonamides and cyclooxygenase inhibiting compounds did not show significant cytotoxic effect against hepatocellular carcinoma, cervix and lung cancer, however, they were much more effective against cell from breast, colorectal and bladder cancers (18). These results are partly in agreement with our data which showed significant differences between the effects of compounds against breast cancer and cervix cell lines (p < 0.001). Regarding to IC_50_ values, tested compounds exhibited a significantly greater cytotoxicity against MDA-MB-468 as breast cancer cells.

From structure activity relationship (SAR) point of view, different groups on the sulfonamides nucleus (R1 and R2 in Table 1) can affect cytotoxicity of compounds. If R2 replaced with H or a halogen (F or Cl), the most cytotoxic compound was obtained. Electron donating groups such as methoxy (-OCH3) and to lesser extent, methyl (-CH3), showed lower cytotoxic effects. However, no significant effects were seen with different R1 groups. Finally, it could be concluded that the order of cytotoxicity of compounds were as followings if R2 replaced with: F or Cl ≥ H > OCH3 > CH3.
